# Subconcussive brain vital signs changes predict head-impact exposure in ice hockey players

**DOI:** 10.1093/braincomms/fcab019

**Published:** 2021-04-06

**Authors:** Shaun D Fickling, Aynsley M Smith, Michael J Stuart, David W Dodick, Kyle Farrell, Sara C Pender, Ryan C N D’Arcy

**Affiliations:** 1 Faculty of Science and Applied Sciences, Simon Fraser University, Metro Vancouver, BC V5A1S6, Canada; 2 Center for Neurology Studies, HealthTech Connex, Metro Vancouver, BC V3V0C6, Canada; 3 BrainNET, Health and Technology District, Surrey, BC V3V0C6, Canada; 4 Department of Physical Medicine and Rehabilitation, Sports Medicine Center, Mayo Clinic, Rochester, MN 55905, USA; 5 Department of Orthopedic Surgery, Sports Medicine Center, Mayo Clinic, Rochester, MN 55905, USA; 6 Department of Neurology, Mayo Clinic, Phoenix, AZ 85259, USA; 7 Creighton University School of Medicine, Omaha, Nebraska 68178, USA; 8 School of Medicine, University College Dublin, Dublin D04 V1W8, Ireland; 9 DM Centre for Brain Health, Radiology, University of British Columbia, Metro Vancouver, BC V6T1Z4, Canada

**Keywords:** concussion, subconcussion, EEG, ERP, brain vital signs

## Abstract

The brain vital signs framework is a portable, objective, neurophysiological evaluation of brain function at point-of-care. We investigated brain vital signs at pre- and post-season for age 14 or under (Bantam) and age 16–20 (Junior-A) male ice hockey players to (i) further investigate previously published brain vital sign results showing subconcussive cognitive deficits and (ii) validate these findings through comparison with head-impact data obtained from instrumented accelerometers. With a longitudinal study design, 23 male ice hockey players in Bantam (*n* = 13; age 13.63 ± 0.62) and Tier II Junior-A (*n* = 10; age 18.62 ± 0.86) divisions were assessed at pre- and post-season. None were diagnosed with a concussion during the season. Cognitive evoked potential measures of Auditory sensation (N100), Basic attention (P300) and Cognitive processing (N400) were analysed as changes in peak amplitudes and latencies (six standard scores total). A regression analysis examined the relationship between brain vital signs and the number of head impacts received during the study season. Significant pre/post differences in brain vital signs were detected for both groups. Bantam and Junior-A players also differed in number of head impacts (Bantam: 32.92 ± 17.68; Junior-A: 195.00 ± 61.08; *P* < 0.001). Importantly, the regression model demonstrated a significant linear relationship between changes in brain vital signs and total head impacts received (*R* = 0.799, *P* = 0.007), with clear differences between the Bantam and Junior-A groups. In the absence of a clinically diagnosed concussion, the brain vital sign changes appear to have demonstrated the compounding effects of repetitive subconcussive impacts. The findings underscored the importance of an objective physiological measure of brain function along the spectrum of concussive impacts.

## Teaser

The brain vital signs framework is a portable, neurophysiological evaluation of brain function. Fickling et al. observed a strong, significant linear relationship between changes in brain vital signs and the total number of subconcussive head impacts sustained over the course of a playing season in male ice hockey players.

## Graphical Abstract

**Graphical Abstract:**
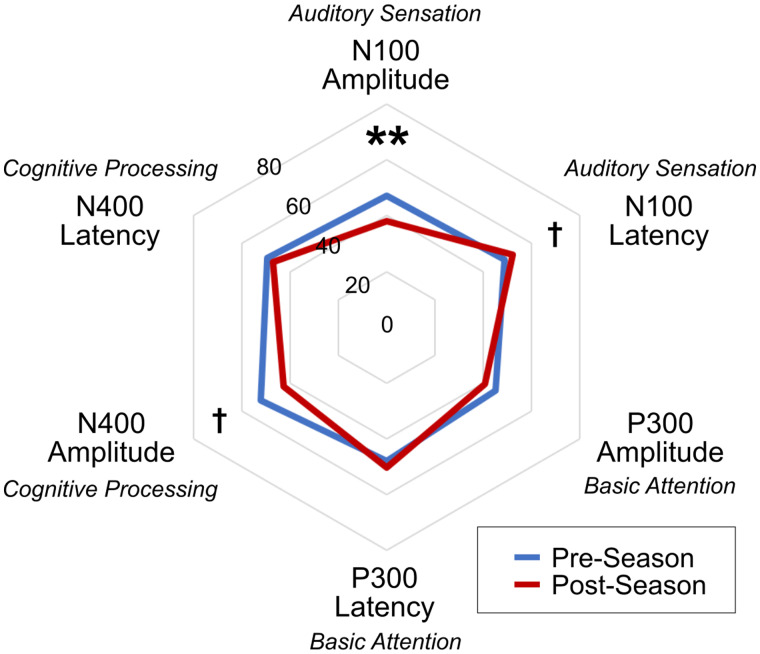

## Introduction

### Sport-related concussion and subconcussion

Scientific evidence on concussion is emerging from studies and discussions involving contact sports such as ice hockey and the concept of cumulative subconcussive impairment is gathering increasing attention.^1^^,^^2^ A subconcussive impact is a mechanical force transmitted to the brain below the threshold for a diagnosis of an acute concussive injury. The effects of these low-magnitude impacts may not even be noticeable to the player or to observers on the sideline. However, it has been shown that repetitive subconcussive impacts over time can result in structural and functional brain impairment.^3^ The extent of impairment is highly correlated with the frequency of exposure to these impacts.^4^^,^^5^

Head impacts in the sport of ice hockey typically result from player-to-player or player-to-surface contact due to body checking, collisions and fighting.^1^ Some of these impacts are the consequence of foul play, but many of these events also result from routine, legal gameplay. The use of head-impact accelerometers provides a portable approach to monitoring the frequency and magnitude of contact that a player experiences during a game or practice.^6^^,^^7^ Head impact acceleration data allow for the evaluation of differences between graduated levels of contact in young players, with anticipated differences between age groups, skill level and experience.

### Objective neurophysiological measures

Objective neurophysiological measures of brain function have demonstrated sensitivity to both concussive and subconcussive impacts.^8–10^ We previously used the brain vital signs framework to provide an objective, accessible, rapid evaluation that is sensitive to changes in individual brain function at the point-of-care.^11–13^ Brain vital signs monitoring uses portable electroencephalography (EEG) as a non-invasive, direct measure of cognitive evoked potentials, also known as event-related potentials (ERPs).^14–16^ ERPs are electrical signals produced by the brain elicited in response to a stimulus. They are frequently used to characterize various attributes of brain function from low-level sensory to high level cognitive processing. ERPs are extracted from raw EEG through signal averaging, in which random EEG activity is averaged to near zero and only non-random EEG signal fluctuations related to specific processing of the stimuli remains. Accordingly, it has been possible to experimentally characterize specific ERP waveform peaks and features as component markers of underlying cognitive brain functions and to track functional changes through changes in timing (latency) and response size (amplitude). ERPs have been successfully implemented in concussion research for decades to detect subtle neurophysiological presentations of brain injury.^17^

Within the brain vital signs framework, well-established ERP responses are obtained using a portable EEG platform.^12^ This approach uses a compressed auditory stimulus sequence, integrating oddball tone stimuli with semantic word pairs to elicit three target ERP components: (i) auditory sensation derived from the N100, (ii) basic attention from the P300 and (iii) cognitive processing from the N400 during semantic language association. The N100^18^ and P300^19^ are derived from the brain’s respective sensory- and attention-based responses to a series of auditory tones in which there are unexpected deviant tones. The N400^20^ is derived from the brain’s response to semantic differences between congruent (‘bread-butter’) and incongruent (‘bread-window’) word pairs. The rationale behind the selection of these three ERP components in the rapid brain vital signs framework is described in detail by Ghosh Hajra et al.^12^ Importantly, they represent among the most well-established responses along the continuum of information processing, from low to higher levels, that can be integrated into a rapid and effective stimulus sequence (6-min scan time).

The ERPs are identified and evaluated for amplitude (synchronous processing) and latency (processing speed), resulting in six total measures. These six measures are subsequently linearly transformed to a standardized score on a 0–100 scale, with larger peak amplitudes and shorter peak latencies resulting in higher scores.^12^ Changes in brain vital sign scores are depicted within a radar plot format, with the transformation process preserving the essential ERP results but enabling practical, simplified interpretation.

Using this framework, we previously demonstrated enhanced sensitivity to subtle changes in brain function in both concussion and subconcussion that were undetected by current clinical concussion protocols.^10^ Fickling et al. monitored brain vital signs in 16–20-year-old male Junior-A hockey players during pre-season, in the early-acute phase of concussion (<24 h), at return-to-play, and post-season.^10^ The findings showed significant early-acute concussive deficits in all brain vital signs measures. Evidence of sustained, significant deficits in basic attention was subsequently also observed in the P300 amplitude despite players being cleared to return-to-play using internationally accepted diagnostic protocols. Importantly, when examining whether deficits accumulated over the season were detectable in players who were not diagnosed with concussion, significant cognitive processing delays were also observed in the N400.

### Objectives and hypothesis

Subconcussive impacts can be further characterized through integrated head-impact data and comparisons between age groups. Accordingly, we monitored brain vital signs in competitive age 14 or under (Bantam) and age 16–20 (Junior-A) ice hockey players before and after a regular hockey season. The objective was to evaluate neurophysiological changes over the season in these age groups and to understand the related effects of cumulative head impacts. The first hypothesis predicted that pre/post-season differences in brain vital sign measures would be present for both age groups, likely due to differences in age, size, speed and playing intensity. The second hypothesis predicted that Bantam players would sustain fewer overall impacts than Junior-A players. The third hypothesis predicted a linear relationship between changes observed in brain vital signs metrics and the number of impacts measured during the playing season across both age groups.

## Materials and methods

### Participants

Institutional ethics boards at Mayo Clinic and Simon Fraser University approved the study. Each participant provided an informed, written consent or assent with parent/guardian consent, according to the declaration of Helsinki. Male players (*N* = 37) were studied in a longitudinal design over a single season of Bantam and Tier II Junior-A level ice hockey. Body checking is permitted in leagues for both age groups. Sixteen male players in the 14 or under (Bantam) age group (*n* = 16) were studied during the 2017–2018 season (mean age: 13.63 ± 0.62 years, height 167.32 ± 10.88 cm, weight 61.66 ± 9.36 kg). Twenty-one male players in the 16–20 age group (Junior-A) players (*n* = 21) were studied during the 2018–2019 season (mean age: 18.62 ± 0.86 years, height 184.69 ± 5.90 cm, weight 85.04 ± 5.85 kg). Players self-reported between 0 and 4 prior concussions (Bantam: mean 0.69 ± 0.98, Junior-A: mean 1.38 ± 1.46) in their playing careers. All reported that they had recovered and were presently asymptomatic at the time of enrolment. Of the entire study group, eight players (one Bantam, seven Junior-A) were diagnosed with a concussion during the study season and excluded from this analysis. Six additional players (two Bantam, four Junior-A) did not complete post-season assessments due to transfers out of the team or withdrawals from the study. All remaining players (*N* = 23: 13 Bantam, 10 Junior-A) were not diagnosed with a concussion during the study season and also completed both pre- and post-season testing. Bantam players competed in 18.77 (± 1.69) home games. Individual participation for Bantam players was only recorded for home games, although the team played 47 total games that season. Junior-A players competed in 45.20 (± 20.21) total games (home 18.19 ± 9.14; away: 21.43 ± 8.23). All participants were required to be fluent in English with normal hearing. Mayo Clinic physicians and athletic trainers provided primary health care for the study participants, including concussion diagnosis and management in accordance with best current clinical practice.

### Data collection

#### Brain vital signs

Brain vital signs baseline testing was completed prior to the start of the hockey season, during pre-season physical training. The duration of the season was ∼5 months for the Bantam players and 7 months for the Junior-A players. Post-season assessments were completed within 1 week of the final game. Players were scanned using a portable 8-channel g. Nautilus EEG system (Gtec Medical Engineering, Austria) at each testing session. Brain vital sign testing incorporated a 5-min automated stimulus sequence consisting of auditory standard-deviant tones interspersed with spoken prime-target word pairs that were either semantically congruent (‘bread-butter’) or incongruent (‘bread-window’). Participants were asked to listen attentively to the stimuli, but no active response was required. To reduce motor and ocular artefacts, participants were instructed to sit motionlessly, and maintain visual fixation on a cross positioned at eye level 2 m away. Distractions were mitigated by performing the scans in a quiet, closed room. The same facility was used for pre- and post-season testing.

One 5-min EEG recording was collected per participant per time point. EEG data were recorded from three midline scalp electrodes (Fz, Cz and Pz) embedded in an elasticized g. Nautilus cap. A reference electrode was clipped to the right earlobe and disposable Ag/AgCl electrodes were used for electro-oculagram recording from the supra-orbital ridge and outer canthus of the left eye. g. GAMMAsys electrode gel was applied to each location to ensure conductivity. Skin-electrode impedances were maintained below the standard 30kΩ requirement at each site.

#### Head impacts

Head impacts were recorded during games using the Triax SIM-G (Triax Technologies Inc., Norwalk, CT, USA) systems for Bantam players and the X-Patch (Prevent Biometrics, Edina, MN, USA) for Junior-A players. Given the different head impact systems, only the number of head impacts per player were analysed to allow for comparisons across groups.

### Data processing

#### Brain vital signs

EEG data were processed in MATLAB (Mathworks, USA). A fourth-order Butterworth filter (0.5–20 Hz) and a custom notch filter (60 Hz) were applied to the raw EEG data. Adaptive filtering^21^ corrected for ocular artefacts as recorded by the electro-oculagram electrodes. ERPs were derived from these representative channels through standard methods of segmentation (range: −100 ms pre-stimulus to 900 ms post-stimulus), baseline correction, and conditional averaging.^16^ A wavelet filter was used to denoise the individual ERP epochs prior to final averaging.^22^ To mitigate any selection bias, ERP peaks were selected with an automated algorithm that chooses the largest local maxima/minima within expected polarities and temporal ranges for the N100 (minima: 80–200 ms), the P300 (maxima: 200–500 ms) and the N400 (minima: 300–700 ms). ERP peaks were then evaluated for both latency (response time) and amplitude (synchronous processing), for a total of six measures. Amplitudes were measured as an adjusted baseline measure relative to the adjacent peaks of opposite polarity. Latencies were defined at the point of peak amplitude. ERP responses were then transformed into standardized brain vital signs scores (0–100), derived from baseline group mean values ±3 standard deviations.^10^ This transformation preserves potential differences in ERP ranges between age groups and provides easily interpretable metrics for evaluating multivariate change-over-time.

#### Head impacts

The total number of impacts received by each player was calculated at the end of the season from the output of the respective accelerometer systems. Other biomechanical measures (impact location, magnitude of linear and rotational accelerations, head injury criterion, etc.) could not be included in this analysis due to potential differences across systems.^6^

### Statistical analysis

Statistical analyses were performed using SPSS (IBM, NY, USA). To assess changes in brain vital signs from pre- to post-season between the age groups, standardized ERP scores were compared using a two-way mixed repeated-measures ANOVA (measurements: six brain vital signs scores; within-subject factors: time; between-subject factors: age group; Greenhouse–Geisser corrected). Differences in the number of head impacts between groups were compared using a two-tailed Wilcoxon rank-sum test. Finally, to assess the relationship between brain vital signs score changes and total impacts, differential (post minus pre) scores were used as the inputs to a ‘best subsets’ automatic linear regression model, which used the adjusted *R*-squared metric to optimize the entry criteria.

### Data availability

The data that support the findings of this study are available on request from the corresponding author.

## Results

### Brain vital signs changes (H1)

**Figure 1:**
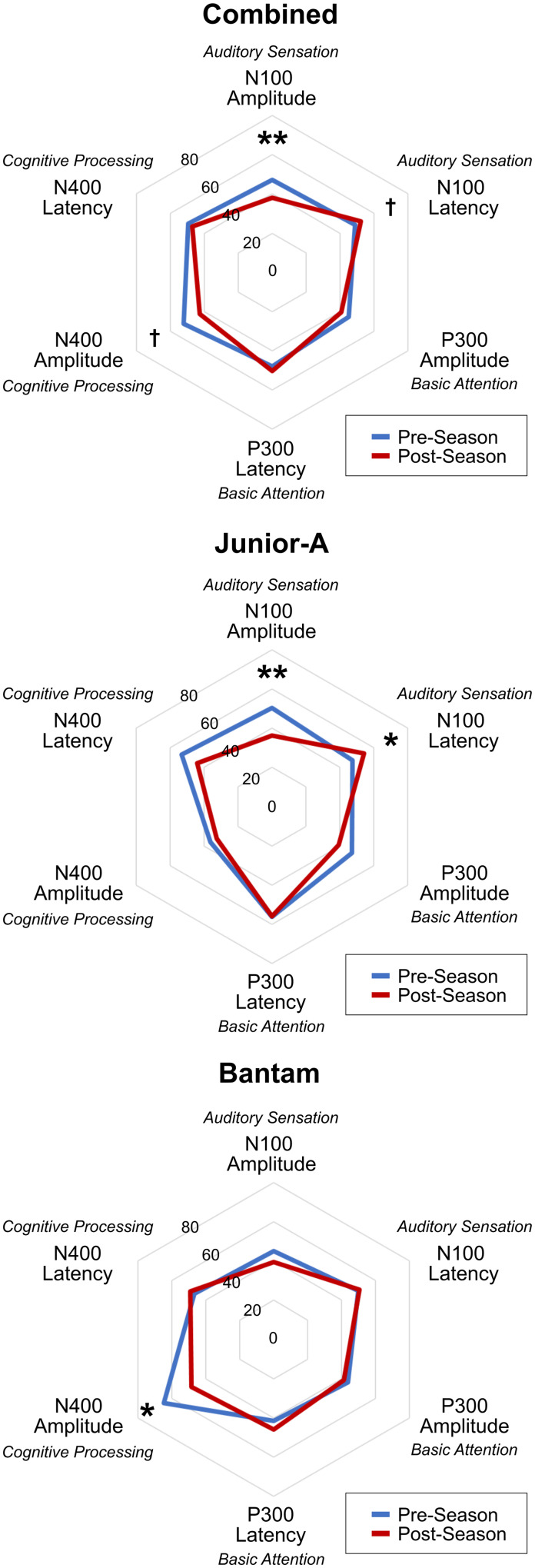
**Radar plots.** Radar plots showing group brain vital signs profile trends from pre-season to post-season, in both groups (top), Junior-A only (middle) and Bantam only (bottom). The combined plot is included to demonstrate the changes across both age groups (*N* = 37). **P < 0.01; *P < 0.05; ^†^*P* < 0.056

**Figure 2:**
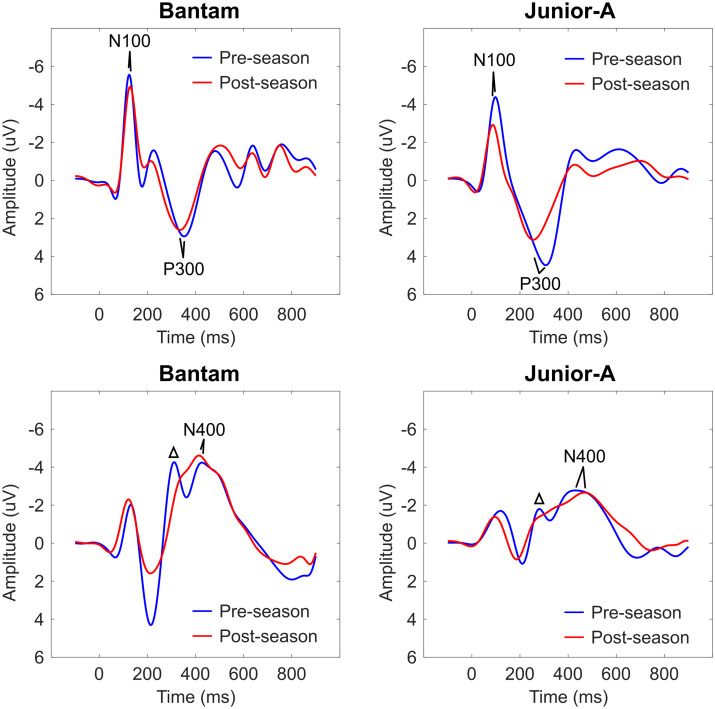
**ERP waveforms.** Group-averaged ERP Waveforms showing Bantam (left column) and Junior-A (right column) groups and brain vital sign responses to Deviant tones (top row) and Incongruent Words (bottom row). Waveform changes in the phonological mismatch negativity (Δ), which precedes the N400.

Figure 1 shows pre/post radar plot differences for each group, as well as a combined radar plot to display the changes that are consistent across both age groups. Figure 2 shows group-averaged ERP waveforms as responses to deviant tone (N100, P300) and incongruent word (N400) stimuli over time are provided for Bantam and Junior-A groups. Representative waveform results of an individual from each group are included in Supplementary Fig. 1. The repeated-measures ANOVA for within-subject effects showed no overall multivariate effect of time (*F*(6,16) = 1.816; *P* = 0.159) or interaction between time and age group (*F*(6,16) = 2.269, *P* = 0.089). A significant overall main effect for time was shown for N100 amplitude (mean change: −9.26 ± 14.31; *F*(1,21) = 10.601; *P* = 0.004). Near-significant main effects for time were shown for N100 latency (mean change: 3.37 ± 8.97; *F*(1,21) = 4.157; *P* = 0.054) and N400 amplitude (mean change: −16.31 ± 21.67; *F*(1,21) = 4.105; *P* = 0.056). *Posthoc t*-tests showed significant within-subject changes across the season for both Bantam (N400 amplitude score decrease: *P* = 0.0229) and Junior-A (N100 amplitude score decrease: *P* = 0.0082; N100 latency score increase: *P* = 0.0139) groups between pre-season and post-season. No significant univariate effects for the interaction between time and age group were found.

The ANOVA measures of between-subject effects showed a significant multivariate effect for age group (*F*(6,16) = 3.997; *P* = 0.012). A significant univariate effect for age group was also found in the N400 amplitude (*F*(1,21) = 15.493; *P* = 0.001), with a trend for P300 latency (*F*(1,21) = 4.112; *P* = 0.055). *Posthoc t*-tests showed a significant difference between groups in the pre-season N400 amplitude measure (Bantam: 64.67 ± 12.41; Junior-A: 36.24 ± 13.02; *P* < 0.0001), while no difference was observed post-season.

### Head impacts (H2)

**Figure 3:**
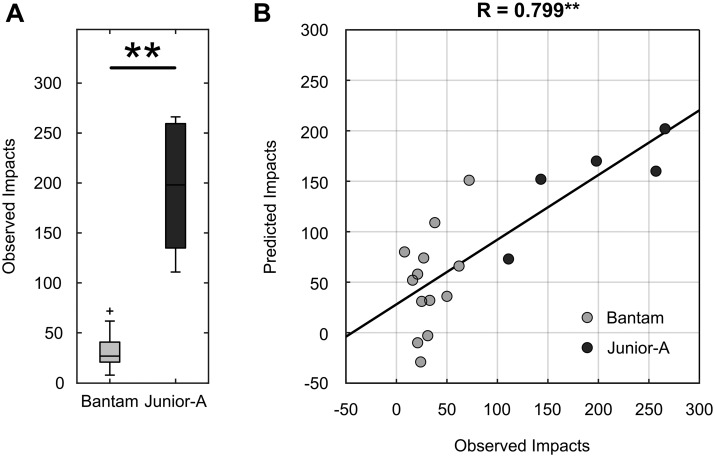
**Head-impact regression model.** (**A**) Differences in number of head impacts received by Bantam (light grey) and Junior-A (dark grey) players during the study season. (**B**) The output of the linear regression model that predicts the number of impacts received based on the changes in brain vital signs over the course of the season. **P < 0.001.

Five of the 10 Junior-A players did not have reliable head impact accelerometer data during the season (<25% usage). These data were excluded from the subsequent analyses. The five Junior-A players who did wear their accelerometers (>80% usage) received significantly more head impacts over the course of the study season than the Bantam group (Bantam: 32.92 ± 18.40, Junior-A: 195.00 ± 68.29; *P* = 0.0002). The differences in number of head impacts sustained during the study season between the two groups are displayed in Fig. 3A.

### Relationship between brain vital signs and head impacts (H3)

Figure 3B shows the observed versus predicted number of impacts across both groups. The automatic linear regression included N100 amplitude, P300 latency, N400 amplitude and N400 latency measures as the best subset of predictors in a model that was able to predict number of impacts received with *R* = 0.799 (*R*^2^ = 0.639, adj. *R*^2^ = 0.528, *P* = 0.007). The details of the regression model are described in Table 1.

**Table 1:** Regression model

Model summary						
*R*	*R* square	Adj. *R* square	*F* change	df1	df2	Sig
0.799	0.639	0.528	5.750	4	13	0.007^**^
	Model coefficients					
	Univariate Pearson correlation		Sig	Regression coefficient	Sig	
Constant term	–		–		0.002**	
N100 amplitude	−0.563		0.008**	−3.991	0.004**	
P300 latency	−0.213		0.198	−1.200	0.167	
N400 amplitude	0.156		0.268	1.851	0.025*	
N400 latency	−0.473		0.024*	−0.750	0.285	

* *P* < 0.05,

** *P* < 0.01. All coefficients that have a significant *P* value are emphasized in bold.

## Discussion

### Main findings

Brain vital sign monitoring detected significant subconcussive pre/post-season effects in both age 14 or under (Bantam) and age 16–20 (Junior-A) ice hockey players. Pre/post-season changes were observed in auditory sensation (N100) and cognitive processing (N400), with differences detected between groups likely related to differences in age, size and playing intensity (Hypothesis 1). These changes can also be observed at the individual level (Supplementary Fig. 1). As predicted, Bantam players had significantly fewer impacts than Junior-A players (Fig. 3A). Importantly, the neurophysiological changes were significantly correlated with the number of head impacts received over the course of the season. Aggregate brain vital sign changes predicted a linear relationship, as high as *R* = 0.799 (*P* < 0.001), between neurophysiological changes and number of impacts (Fig. 3B).

### Subconcussion

The neurophysiological effects of subconcussive impacts on the brain were not extensively studied prior to 2010. However, recent research has focused on the role of these impacts in both short- and long-term measures of brain function. There is evidence to suggest that even a single practice session involving head contact, such as heading a ball in soccer, can result in impairment.^23^ Magnetic resonance imaging studies have demonstrated that players exposed to repetitive head impacts over the course of a season show significant abnormalities compared to controls over the same time period and that these abnormalities are related to the extent of exposure to head impacts.^4^^,^^5^^,^^24^

EEG rapidly records individual neurophysiological ERP responses and is well suited for assessments directly at the side of a rink or field.^10^^,^^25^ Prior cognitive ERP research in players with accumulated subconcussive exposure have demonstrated P300 amplitude reductions after a season of sport.^26^^,^^27^ Similarly, we previously reported a significant N400 latency effect in the absence of a diagnosed concussion in another group of Junior-A ice hockey players.^10^

With the age range expanded to combine and compare Bantam and Junior-A, it was possible to evaluate whether meaningful patterns of subconcussive changes emerge (Fig. 2). While preliminary, the N400 differences prompt continued exploration of cognitive processing changes in contact sport players. Waveform morphology differences suggested that subconcussive changes may also be associated with changes in earlier processing, such as perceptual and phonological processing, in addition to semantic cognitive processing changes (Fig. 2). Specifically, future investigations into possible pre/post changes in the well-established components like the P200/P300 and phonological mismatch negativity that immediately precede the semantic N400 are warranted.^28^^,^^29^ The N100 differences further support the concept of complex cognitive changes resulting, in part, from earlier, lower-level deficits in sensory attentional processing.^30^

While overall subconcussive patterns may emerge, direct comparisons between studies are challenging. It is unlikely that there is one single presentation of subconcussive impairment. Cumulative exposure to head impacts is thought to result from general structural disruption of axonal white-matter tissue.^31^ The resulting presentation will likely vary in terms of specific neurophysiological presentation depending on where in the brain these micro-injuries occur. It will therefore be important to link functional measures of subconcussive impairment to abnormalities in structural imaging, functional/clinical outcomes and key biomechanical factors such as head-impact exposure.

### Head impacts

The use of accelerometers to measure head impact biomechanics has shown to have variable reliability across different sensor designs, given that accelerations relative to a helmet or skin-patch do not necessarily directly correlate to accelerations in the brain.^6^^,^^32^ Furthermore, accelerometer error can also be attributed to sensor placement, adhesion, skin movement, interactions with equipment, etc.^6^ Video analysis can be used to confirm that an impact was accurately recorded, although this is both time-consuming and resource intensive. Promising research is ongoing that combines multi-camera-angle video recordings to create impact models to estimate the biomechanics of injury.^33^^,^^34^

Due to this uncertainty, instrumented accelerometers without video-analysis validation may not always fully capture the specific biomechanics of individual impact events. However, portable sensors can approximate cumulative head-impact measures over a time period, particularly at the group level. If the sensor variance is equivalent for all players, then measures of central tendency or cumulative measures from accelerometer data are practical measures of head-impact exposure. Recording individual playing time or number of games played are helpful in terms of exposure to the risk of concussion but do not which account for differences in playing style, penalties, position, size, speed, etc.

### Differences between age groups

ANOVA detailed a significant overall effect for age group, specifically in the N400 amplitude component. *Posthoc t*-tests revealed a significant difference between groups in the pre-season N400 amplitude measure, where Bantam players had larger N400 amplitudes, but this difference was no longer present at post-season. This finding was attributed to a significant decrease in N400 amplitude across the season in the Bantam group (Figs 1 and 2), which was not observed in the Junior-A group. These baseline differences suggest a potential cumulative effect of impacts over a longer duration, and the pre–post differences between groups suggest differential effects on cognitive processing due to cognitive and biomechanical factors related to age.

There was also a significant N400 latency correlation with number of impacts across both groups (Table 1). This suggests that N400 changes, particularly latency delays, may provide a potential indicator of severity for subconcussive impacts. Given previous findings showing N400 latency changes in Junior-A players,^10^ future studies should further examine whether N400 peak latency measures can be used to provide both sensitivity and specificity for deficits along the subconcussive-to-concussive continuum of injury. Additional work should investigate which electrophysiological measures relate to functional outcomes.

### Caveats

Sport-related concussion research is often challenged in terms of sample size and representation along with technical implementation limitations. The current findings are based on a sample of male ice hockey players and therefore may not generalize to other populations. Future studies will expand the scope to include both male and female players from a range of sports (both contact and non-contact), age groups and divisions as well as non-athlete control populations for comparison. The repeated-measures statistical model allows for each player to act as their own control. However, incorporating additional matched control groups for comparison would further identify changes related to head impact biomechanics in future studies. Also, players should be assessed more frequently during the season to better characterize a profile of subconcussive changes. Assessments before and after individual games will be important to further integrate measures of head impact exposure, and external validation can be accomplished by video analysis. Furthermore, accelerometers were only worn during games for this study, so it is unclear how contact at practices influenced the results. Different types of accelerometer systems were used between the groups due to performance and compliance issues. While this may have resulted in minor differences in measurement of head impacts, the analysis was restricted to number of impacts to avoid this methodological complication. Nonetheless, compliance among players was challenging in terms of consistent accelerometer measurements during games and across the season. As such, the linear regression model was used with both groups. With improved accelerometer technology and compliance, the relationship(s) between brain vital sign changes and head impact biomechanics as a factor of age group can be better characterized.

Prior concussion history was evaluated but not included in this analysis; future work should include number of previous concussions as a covariate to determine if this influences outcomes for subconcussive and/or concussive impacts. A final caveat is the lack of correlation with existing objective functional outcome measures such as balance, cognitive, oculomotor, affective/mood, sleep, etc. Provided that objective neurophysiological changes have greater sensitivity, future work should focus on relating brain vital signs changes to these objective clinical outcome measures. This will help to determine the extent to which the changes correlate with functional outcomes for diagnosis and to predict recovery.

## Clinical relevance and conclusions

As expressed by Nauman and Talavage,^35^ it is increasingly important that the general understanding of concussion shifts from a singular acute-injury model to a spectrum of head-impact exposure and related outcomes. Objective measures of brain function can more accurately assess subtle changes along this spectrum. Brain vital signs were sensitive to the effects of repetitive subconcussive impacts in Bantam and Junior-A ice hockey players across a season, specifically detecting significant changes both within and between groups that were directly related to the number of impacts. Observed changes in the N400 represent more complex mechanisms in cognitive speech processing that should be explored further. The findings expand upon previous work by linking brain vital signs changes over time to the extent of exposure to head impacts.

## Supplementary Material

fcab019_Supplementary_Data

## Abbreviations

EEG =: electroencephalography
ERPs =: event-related potentials
