# Supplementary material for: Subconcussive brain vital signs changes predict head-impact exposure in ice hockey players
Source: Brain Commun. 2021 Apr 6;3(2):fcab019. doi: 10.1093/braincomms/fcab019 (PMC8023684; doi:10.1093/braincomms/fcab019)
Supplement: fcab019_Supplementary_Data [file fcab019_supplementary_data.docx]

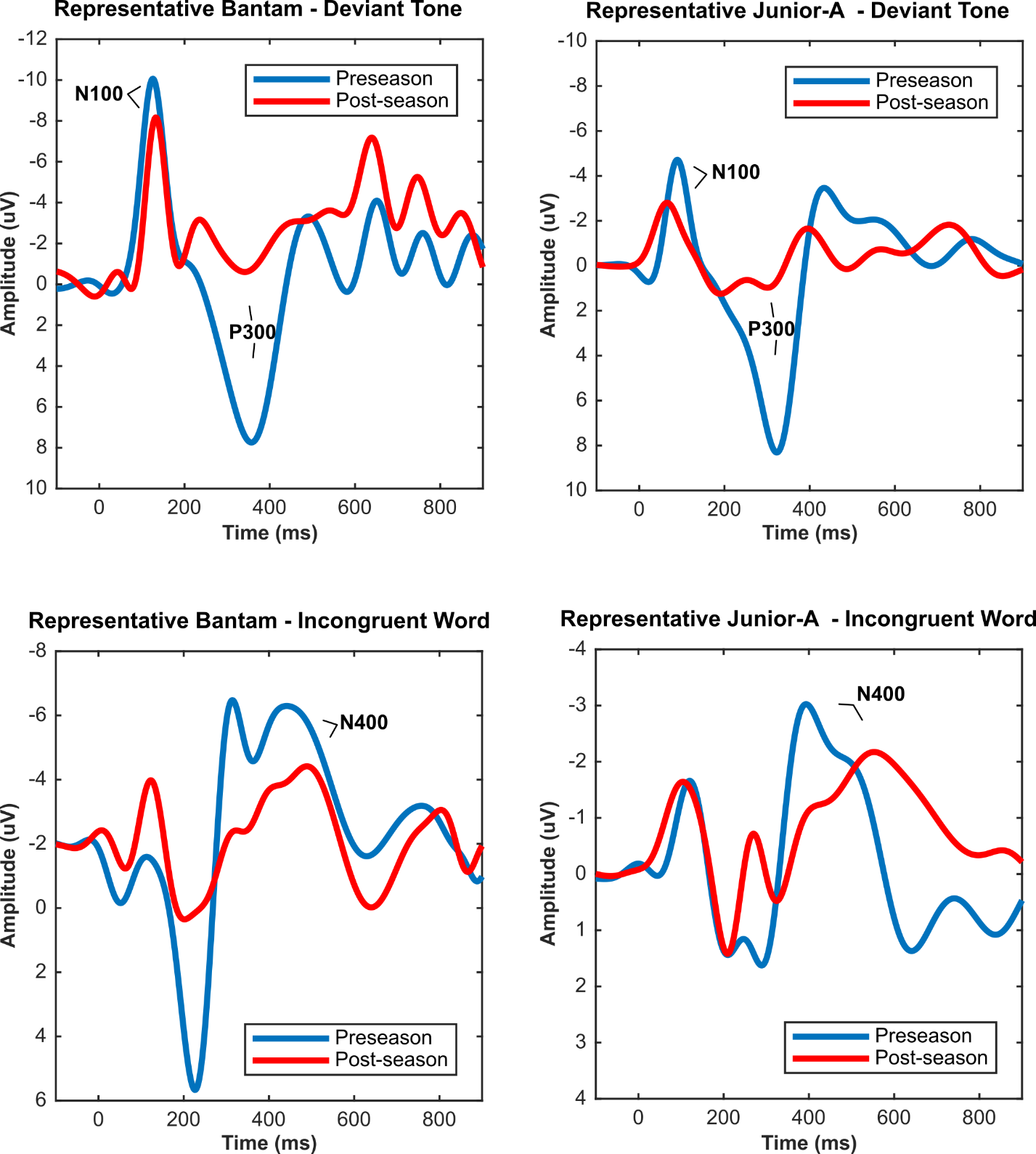


Supplemental Figure 1: Representative waveforms for each age group showing changes at the individual level
